# Lower limb function and quality of life after ILP for soft-tissue sarcoma

**DOI:** 10.1186/s12957-017-1150-3

**Published:** 2017-04-13

**Authors:** Lars Erik Podleska, Nevda Kaya, Farhad Farzaliyev, Christoph Pöttgen, Sebastian Bauer, Georg Taeger

**Affiliations:** 1 Sarcoma Surgery Division, Department of General, Visceral and Transplantation Surgery, University Hospital of Essen and Sarcoma Center at the West German Cancer Center (WTZ), University of Duisburg-Essen, Hufelandstr. 55, 45147 Essen, Germany; 2 Department of Radiotherapy, University Hospital of Essen and Sarcoma Center at the West German Cancer Center (WTZ), University of Duisburg-Essen, 45147 Essen, Germany; 3 Department of Medical Oncology, University Hospital of Essen and Sarcoma Center at the West German Cancer Center (WTZ), University of Duisburg-Essen, 45147 Essen, Germany

**Keywords:** Isolated limb perfusion, TNF-alpha, Melphalan, Soft-tissue sarcoma, Quality of life, Limb function, Long-term survival

## Abstract

**Background:**

Isolated limb perfusion with TNF-alpha and melphalan (TM-ILP) in combination with complete tumor resection is an effective treatment option for non-resectable soft-tissue sarcoma of the extremities, with limb salvage rates greater than 80%. The aim of this study was to assess quality of life (QoL) after TM-ILP, also with regard to long-term survival.

**Methods:**

We retrospectively examined 27 patients who had primarily non-resectable soft-tissue sarcoma of the leg and who had undergone TM-ILP and complete tumor resection (with limb-sparing intent) during their follow-up examinations using the Quality of Life Questionnaire (QLQ-C30) and the German Short Musculoskeletal Function Assessment (SMFA-D). The results from the QLQ-C30 were compared to the reference values for the general population, to the “all cancer patients” reference values (both reference values published by the European Organization for Research and Treatment of Cancer (EORTC)), and to the reference values of a historical amputation group from the literature. The results of the SMFA were compared with those from a reference group of healthy individuals.

**Results:**

Surprisingly, we found that the global health status/QoL in the TM-ILP group was not significantly different from the general population or from patients with amputation, but it was higher than that of patients with cancer in general. Concerning the SMFA, we did find functional impairments in patients after TM-ILP compared to the reference group. With regard to long-term survival, we found no time-dependent deterioration in QoL for longer time intervals after treatment.

**Conclusions:**

These results support the use of TM-ILP in limb-sparing multimodal therapy settings from a quality-of-life perspective, but they also encourage further research on this matter.

**Electronic supplementary material:**

The online version of this article (doi:10.1186/s12957-017-1150-3) contains supplementary material, which is available to authorized users.

## Background

Assessment of a patient’s quality of life (QoL) has become a key issue in cancer treatment over the past two decades. The fundamental underlying question is the extent to which QoL is ultimately affected by the treatment of the tumor [1]. For the treatment of soft-tissue and bone sarcoma of the extremities, this aspect becomes essential with respect to determining whether to pursue limb salvage or to select an amputation.

Particularly for large, primarily non-resectable STS (soft-tissue sarcoma) of the extremities, amputation still appears to be a treatment option that is regularly offered to patients, although modern multidisciplinary treatment concepts could often save the patient’s limb. One of these treatment modalities is TM-ILP (TNF-melphalan-based isolated limb perfusion), which has developed into a successful treatment option for primarily non-resectable soft-tissue sarcoma of the extremities. The response rates for TM-ILP range between 60 and 80%. Limb salvage rates for tumors with a primary indication for amputation or at least mutilating surgery (including the resection of major nerves and vascular structures with no adequate means of restoring limb function) are well above 80%, as reported by all high-volume centers performing TM-ILP [2–8]. Pretreatment with TM-ILP leads to devitalization of the tumor’s margins and stabilization of the fibrous tumor capsule [9, 10], which allows for safe tumor resections with close or even marginal resection margins and thus successful limb salvage [11] in otherwise primarily non-resectable STS. The term “non-resectable” refers to tumors for which oncologic resection would result in amputation or the resection of major nerves and/or vascular structures that would lead to major functional morbidity, as stated by Eggermont et al. in the initial TM-ILP study [12].

A number of QoL studies have focused on sarcoma patients. Some of these studies consider sarcoma subgroups, such as patients with metastasized disease [13], certain surgical techniques [14], different body regions [15, 16], or different radiotherapy regimens [17]. Tang et al. [18] performed a systematic review of QoL studies concerning sarcoma patients. They concluded that extremity function, social function, and pain in sarcoma survivors were worse than in the general population [19–21]. On the other hand, several studies showed that patients who underwent amputation instead of limb-sparing surgery had a worse functional outcome [19–23].

Thijssens et al. [21] found these deficiencies in a group of patients after TM-ILP and complete tumor resection. TM-ILP itself can have severe short-term side effects ranging from postoperative swelling and reddening to toxic compartment syndrome and even therapy-associated limb loss (with a risk <1%) [24, 25]. Long-term side effects, such as neuropraxia or vascular complications caused by TM-ILP, in addition to the functional impairment caused by the tumor resection, are found in almost 10% of patients [7].

The aim of the current study was to gain greater and deeper insight into patients’ QoL after TM-ILP and complete tumor resection in a limb-sparing therapy concept, with a secondary focus on patients with long-term survival after soft-tissue sarcoma.

## Methods

**Fig. 1:**
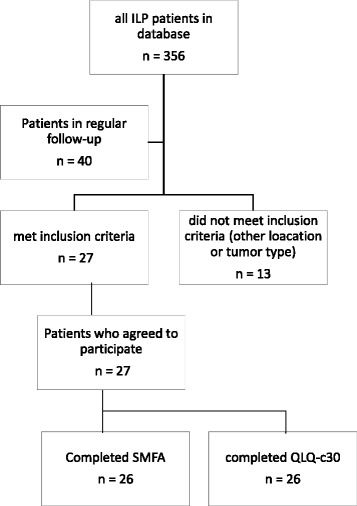
Flow chart presenting the TM-ILP patient selection process

Patients were recruited for this retrospective QoL survey between April 2014 and December 2015 during their follow-up appointments at our outpatient clinic. Figure 1 shows a flow chart of the patient selection process. All patients included in this analysis were previously treated due to non-resectable soft-tissue sarcoma of the lower extremity (according to the definition of Eggermont et al. [12]). Patients were selected for this QoL analysis when they met the following criteria:

- Initial TNF-melphalan-based isolated limb perfusion (TM-ILP)
- Complete resection of the residual tumor after TM-ILP
- Not being under any further treatment regimen (e.g., radiation therapy or chemotherapy) at the time of the test

All patients who met the inclusion criteria and presented themselves at the follow-up appointment were asked whether they would participate in this strictly voluntary survey consisting of two questionnaires. This study was approved by the university’s ethics committee and was conducted in accordance with the Declaration of Helsinki.

### Treatment concept for non-resectable STS

By definition, TM-ILP itself cannot be considered a curative treatment; however, the combination of TM-ILP followed by resection of the residual tumor can establish local tumor control and, thus, limb salvage in more than 80% of cases [2–7]. Therefore, it can be a useful part of a multidisciplinary treatment regime with curative intention. All the patients in this study were treated with primarily curative and limb-sparing intent. Therefore, all the patients underwent TNF-melphalan-based isolated limb perfusion based on the inclusion criteria initially described by Eggermont et al. [12] (primarily non-resectable soft-tissue sarcoma with an indication for amputation or substantially mutilating resections, such as resection of major nerves and vessels). TM-ILP was performed in a standardized manner as previously described [26]. Further explanation and technical details are provided in Additional file 1 linked to this article.

Six weeks after TM-ILP, the response of the tumor to ILP was assessed using MRI, and the remaining tumor was removed with close margins (usually 1–2 mm safety margin) in a limb-sparing and function-preserving resection. Tumors were assessed for treatment response and resection margins. All patients were offered additional postoperative radiotherapy. In cases of a poor response to ILP treatment (>50% vital tumor according to pathologic assessment), patients were strongly recommended to undergo postoperative radiotherapy with a dose of up to 60 Gy in conventional fractionation.

### Quality-of-life assessment: QLQ-C30

Two different scoring systems were used in this study. To examine the patient’s general QoL, we used the QLQ-C30 developed by the European Organization for Research and Treatment of Cancer (EORTC) [27, 28]. This protocol was established in 1993 with the goal of assessing the health-related QoL of patients participating in clinical trials for the treatment of cancer. The advantages of the QLQ-C30 are that it is well established and there are reference values facilitating comparisons of patients with different types of cancer and healthy volunteers. The questionnaire and the scoring system for this study were obtained from the EORTC, and we used the latest version of the questionnaire (version 3.0).

The QLQ-C30 [27] contains 30 questions and consists of the following:

- One *global health status/QoL* scale (health- or disease-related QoL)
- Five function scales (physical, role, emotional, cognitive, and social functioning)
- Nine symptom scales (fatigue, nausea/vomiting, pain, dyspnea, insomnia, appetite loss, constipation, diarrhea, and financial difficulties)

Questions 1 through 28 vary from 1 to 4 points (range = 3), while the last 2 questions, which concern the global health of the patients, vary from 1 to 7 (range = 6). For this analysis, the results were transformed to a scale ranging from 0 to 100. For the global health (QoL) scale and the five function scales, a higher value represents a better QoL or better function, while for the nine symptom scales, a higher value represents a higher level of symptoms/problems.

#### Comparison of patients with short- and long-term follow-up

To assess whether function and QoL in patients with long-term survival (in this case, patients with longer follow-up intervals) are different from those of patients with shorter follow-up intervals, we split our patient cohort into two groups: patients with less than and patients with more than 3 years of follow-up time (interval from TM-ILP to analysis) and compared both groups’ QLQ-C30 and SMFA scales. The rationale for a division after 3 years of follow-up was that more than 85% of local recurrence and metastasis in STS occurs within the first 3 years after diagnosis [29, 30], and our patient cohort was divided equally between these two groups.

#### Reference values for the QLQ-C30 global health status/QoL

To compare the results from the QLQ-C30 global health status/QoL scale, we used the EORTC’s reference values for the *general population* and for *all cancer patients* [31] because these values include no specific reference values for musculoskeletal tumors or sarcoma. The EORTC’s reference values contain case numbers, means, and standard deviations. Because the alternative to TM-ILP and complete tumor resection would have been amputation of the affected extremity, we also used a historical group of sarcoma patients who had undergone amputation due to extremity tumors [23]; these patients had been examined using the same QLQ-C30 questionnaire. The study provided the means, standard deviations, and numbers of patients treated for the global health status/QoL scale of the QLQ-C30.

### SMFA-D

In addition to QoL, we aimed to provide a more precise picture of the possible dysfunctions patients experienced after TM-ILP treatment and complete tumor resection. Because TM-ILP was used for the treatment of tumors at different levels of the leg, we selected a second scoring system to measure patients’ handicap and dysfunction of the extremities. The Short Musculoskeletal Function Assessment (SMFA) is a questionnaire that consists of 46 items addressing the function of the musculoskeletal apparatus on different subscales [32] and is derived from the longer MFA, which was developed in 1996 [33]. In 2000, a German translation (SMFA-D) was developed and tested [34, 35]. The advantage of the SMFA-D over general QoL questionnaires, such as the QLQ-C30 or the SF-36, is its specific orientation toward an orthopedic patient group [36].

The SMFA [32] consists of two scales: the dysfunction index and the bother index. The dysfunction index contains 34 items that are answered on a 5-point scale: 1 point for good and 5 points for poor function. The items on the dysfunction scale can be grouped into four subcategories (mobility, function of the arm and hand, daily activities, and emotional status). The results are then transformed into a scale ranging from 0 (best function) to 100 (poorest function). The bother index consists of 12 items that are also answered on a 5-point scale; these results are also transformed into a scale ranging from 0 (not bothered at all) to 100 (extremely bothered).

#### Reference values for SMFA-D

To interpret the results from the SMFA-D, we used reference values. As no comparable historical data for the SMFA-D were available, we collected data from a group of healthy working volunteers.

### Statistics

The evaluation of the data was performed using SPSS 24 (IBM, New York, NY, USA). The values presented in the results section represent the means ± standard deviation (SD). Figures 3, 4, and 5 show the means and 95% confidence intervals as whiskers on the bar graphs. Two-sample *t* tests were used to test for significance of differences in global health status/QoL between TM-ILP patients, the EORTC’s reference groups, and the amputation group. To correct for cumulative α-error in multiple testing, we applied the Holm-Bonferroni method on the results of the *t* test.

For comparisons of the short- and long-term follow-up groups and of the SMFA TM-ILP patients and the SMFA reference group, we applied the Mann-Whitney *U* test. For all significance tests, the level of significance was defined as *p* < 0.05.

## Results

### Patient characteristics

Twenty-seven patients were enrolled in this study, including 14 males and 13 females with a mean age of 52.7 years, ranging from 12 to 73 years. The mean interval from TM-ILP to participation in this study was 37 ± 26 months and ranged from 5 to 111 months. Figure 1 shows the patient selection process. Forty patients were receiving regular follow-up care at our outpatient clinic, and 27 of these met the inclusion criteria. One patient completed only the SMFA, and one patient failed to answer the general health status/QoL questions of the QLQ-C30, leaving 26 fully completed questionnaires in each group. The incomplete QLQ-C30 questionnaire was evaluated on all other scales but the general health status/QoL scale.

### QLQ-C30: short- and long-term follow-up groups

There were 14 patients (8 males, 6 females) in the short-term follow-up group (less than 36 months) and 13 patients (6 males and 7 females) in the long-term-follow-up group (more than 36 months). Patients in the long-term follow-up group were older (58 ± 11 years) than the patients in the short-term follow-up group (47 ± 18.2 years). The mean follow-up interval in the short-term follow-up group (< 36 months) was 17 ± 8.2 months. The mean follow-up-interval in the long-term-follow-up group (≤ 36 months) was 58 ± 22.9 months.

### Demographic Characteristics of QLQ-C30 reference groups

The *general population* group of the EORTC consisted of 7802 individuals with a mean age of 47.2 years, including 52% males and 48% females. The all cancer patients group from the EORTC included 23,553 patients with a mean age of 58.6 years. This group comprised 56% male and 38% female patients; 6% of the patients had missing gender data.

The patients in the historical amputation group from Zahlten-Hinguranage et al. [23] were younger (median age at examination, 37 years; range, 15–76 years) than the ILP patients. There were 15 males (68%) and 7 females (32%) in this group.

### Characteristics of the SMFA-D reference group

The SMFA reference group contained 12 individuals. The mean age was 50 ± 14.9 years, ranging from 28 to 74 years. There were four males and eight females in this reference group.

### Tumor entities, histopathologic response to TM-ILP, radiotherapy and local recurrence, time after TM-ILP

The most common tumor entity in this patient group was undifferentiated pleomorphic sarcoma, which occurred in nine cases. There were seven cases of liposarcoma (five myxoid and two dedifferentiated liposarcoma) and three cases of synovial sarcoma (Table 1). Less common were myxoid fibrosarcoma and leiomyosarcoma (two cases each). The rare entities in this group involved single cases of clear-cell sarcoma, one epithelioid sarcoma, a malignant peripheral nerve sheath tumor (MPNST), and a dedifferentiated extraskeletal chondrosarcoma. There were 6 iliac and 21 femoral perfusions. Twenty-one patients had primary tumors, four patients were treated for the first local recurrence, and two patients were treated for the second or more local recurrence.

**Table 1:** Characteristics of the TM-ILP group

	Number	Percent
Total	27	100
Tumor entity		
Undifferentiated pleomorphic sarcoma	9	33.3
Myxoid liposarcoma	5	18.5
Synovial sarcoma	3	11.1
Leiomyosarcoma	2	7.4
Dedifferentiated liposarcoma	2	7.4
Myxoid fibrosarcoma	2	7.4
MPNST	1	3.7
Dedifferentiated chondrosarcoma (extraskeletal)	1	3.7
Epithelioid sarcoma	1	3.7
Clear-cell sarcoma	1	3.7
ILP vascular access level		
Iliac	6	22
Femoral	21	78
Grade of regression according to Salzer-Kuntschik		
°1	6	22.2
°2	2	7.4
°3	7	25.9
°4	4	14.8
°5	6	22.2
°6	1	3.7
Missing	1	3.7
Local recurrence (LR) after TM-ILP		
No LR	21	77.8
LR after TM-ILP	6	22.2
Treatment due to primary tumor or local recurrence		
Primary tumor	21	77.8
1st recurrence	4	14.8
>1st recurrence	2	7.4
Radiotherapy (RT)		
No RT	10	37
Postoperative RT	15	55.6
RT not related to QoL analysis	2	7.4

Tumor and treatment-related characteristics of the TM-ILP group

Seven patients (26%) had an unfavorable response, with greater than 50% of viable tumor remaining after TM-ILP, while 73% were considered treatment responders according to the definition published by Deroose et al. [11]. One resection specimen (3.7%) could not be properly assessed for treatment response because primary histopathology was unavailable for a comparison of tumor necrosis and sclerosis. There were six cases of local recurrence (22.2%) after TM-ILP.

### QLQ-C30: global health status/QoL

**Fig. 2:**
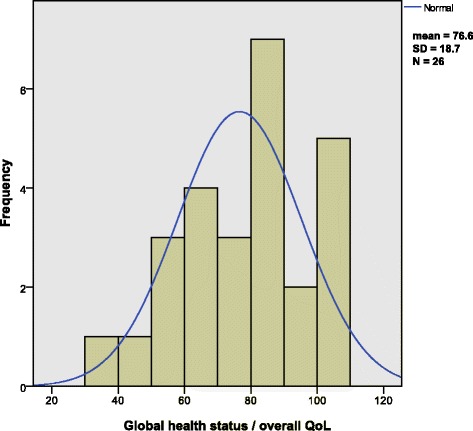
Histogram displaying the distribution of overall QoL scores in the TM-ILP patient group. Overlying is the normal distribution curve

Figure 2 contains a histogram showing the distribution of the values from the QLQ-C30 global health status/QoL of the TM-ILP patient group**.** The sample distribution is somewhat skewed, but the *t* test applied is known to be robust against such a deviation from normality, especially since there are no outliers. Table 2 compares these results with the EORTC “general population,” the EORTC “all cancer patients” [31], and the historical amputation group reported by Zahlten-Hinguranage et al. [23]. Importantly, the QLQ-C30 function scales range from 0 to 100, with higher scores representing better function.

**Table 2:** Comparison of the QLQ-c30 overall QoL/general health scale scores of TM-ILP patients and three reference cohorts

	TM-ILP patients	Amputation [23]	General population [31]	All cancer patients [31]
Mean	*76.6*	69	71.2	61.3*
SD	*18.7*	11	22.4	24.2
*N*	*26*	22	7802	23,553
Margin of error (5%)	*7.6*	4.9	0.5	0.3
95% CI (lower)	*84.2*	73.9	71.7	61.6
95% CI (upper)	*69.0*	64.1	70.7	61.0
*p* (2-sample *t* test vs. TM-ILP patients)		0.1007	0.2196	0.0013
Order of *p* low to high (Bonferroni-Holm)		2	3	1
Comparison to Bonferroni-Holm-corrected alpha-level		0.025	0.05	0.0167
Result of significance testing according to Bonferroni-Holm		n.s.	n.s.	Significant
Effect size (Cohen’s *d*)		0.49	0.24	0.63

*n.s.* not significant, *indicates significance compared to TM-ILP patients

Global health status/QoL, as measured with the QLQ-C30, was not significantly different between the patients in the TM-ILP group (76.6 ± 18.7), the general population (71.2 ± 22.4), and the amputation group (69 ± 22.2). However, compared to the reference values of the all cancer patients group (61.3 ± 24.2), the scores of the TM-ILP patients were significantly higher. The effect size according to Cohen’s *d* shows a medium effect (*d* = 0.63).

### Comparison of short-term vs. long-term-follow-up patients

**Fig. 3:**
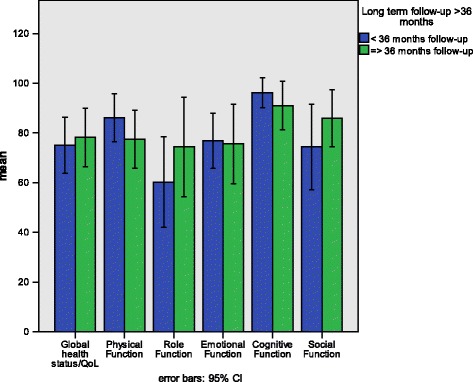
Means and 95% confidence intervals comparing the QLQ-C30 function scale scores of TM-ILP patients with short-term and long-term follow-up after treatment

**Fig. 4:**
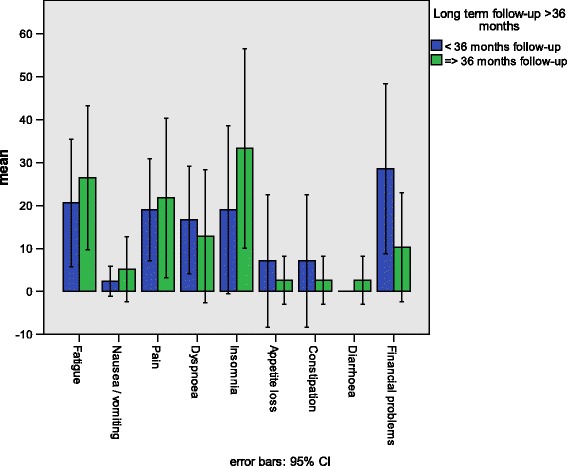
Means and 95% confidence intervals comparing the QLQ-C30 symptom scale scores of TM-ILP patients with short-term and long-term follow-up after treatment

Figure 3 shows the function scales of the QLQ-C30 comparing patients with short-term and long-term follow-up. There were no significant differences between the patients with a follow-up of less than 36 months (ST-FU), and the patients with more than 36 months follow-up (LT-FU) on the general QoL/global health scale or any of the function scales. Figure 4 shows the symptom scales of the QLQ-C30. Again, we found no significant differences between the ST-FU and LT-FU groups. Furthermore, the analysis of the SMFA-D revealed no significant differences between the ST-FU and LT-FU groups on any of the SMFA-D scales (data not shown).

### SMFA-D vs. general population

**Fig. 5:**
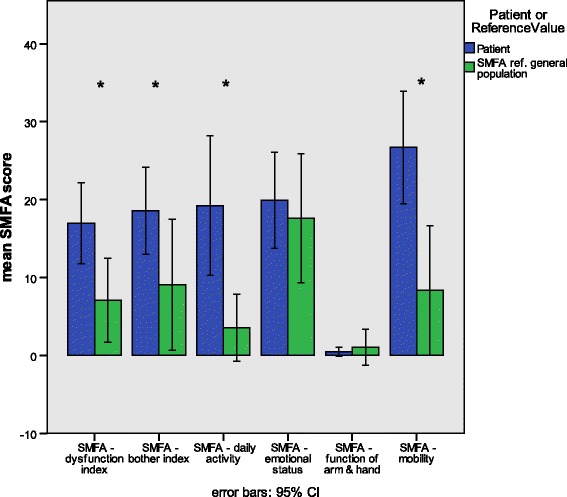
Means and 95% confidence intervals comparing the SMFA scale scores of TM-ILP patients (*patients*) to those of a reference group of healthy volunteers (*SMFA ref. general population*). Significance was calculated with the Mann-Whitney *U* test and is indicated by * (*p* < 0.05)

The interpretation of the SMFA scales is similar to that of the QLQ-C30 symptom scales: higher scores represent a higher degree of the problem or dysfunction. Figure 5 shows the results from the SMFA comparing the TM-ILP patients and a group of healthy volunteers. The first item shown is the SMFA dysfunction index, which was significantly higher (*p* < 0.05) for the TM-ILP patients (16.9 ± 12.9) than for the healthy reference group (7.1 ± 8.5). The *bother index* was significantly higher for the TM-ILP patients (18.6 ± 13.9) compared to the reference group (9.0 ± 13.2). On the daily activity scale, the TM-ILP patients showed significantly (*p* < 0.05) more problems (19.2 ± 22.2) than the reference group (3.5 ± 6.8). There was, however, no difference in the emotional status scale between the TM-ILP patients (19.9 ± 15.3) and the reference group (17.6 ± 13.0). For the function of arm and hand scale, there was no significant difference between the TM-ILP patients, who had almost no symptoms at all (0.5 ± 1.5) and the reference group (1.0 ± 3.6). Finally, regarding the mobility scale, the TM-ILP patients (26.7 ± 17.9) did show a significantly higher (*p* < 0.05) grade of impairment than the reference group (8.3 ± 13.1).

## Discussion

This study aimed to determine how health-related QoL and limb function were affected by multimodal treatment, which was in this case a combination of initial TM-ILP and complete resection of the residual tumor, in patients with primarily non-resectable soft-tissue sarcoma of the extremities. Based on the assumption that patient QoL was most affected by limb impairment, we used two separate scoring systems: first, a general QoL scoring system (QLQ-C30) and second, a more limb function-oriented scoring system (SMFA).

When comparing patient QoL after limb-sparing multimodal therapy with TM-ILP to the QoL among the general population, we found no difference in the global health status/QoL of the QLQ-C30. This finding was somewhat surprising, as one would expect the ILP patient group to show impairments in QoL compared to the general population. One possible explanation for this phenomenon might be that the patients had learned to live with their disability and that there was an overlying *response shift* effect. In fact, the response shift phenomenon has been described as a possible disturbing factor in QoL analyses [37, 38], which can lead to unpredictable outcomes because patients do adapt their life goals according to achievability, which can be lowered over time due to a cancer diagnosis, for example [39]. Of course, a second possibility is type II error, in which the statistical analysis fails to positively identify a difference between the groups. Different authors have described limitations in the QoL of sarcoma patients, especially on the physical and role function scales of the SF-36 [19–21], which suggests type II error as a possible reason for the lack of difference in the QoL scale in our analysis. On the other hand, Porzsolt et al. [40] found that the overall QoL scale of the QLQ-C30 was not adequately represented on the SF-36. Gill and Feinstein [41] stated that QoL is much more dependent on the patient’s own perception than what many function-orientated QoL scales can reveal. Therefore, it seems reasonable to assume that the global health status/QoL scale of the QLQ-C30 is more prone to the response shift phenomenon than any of the function-orientated scales.

The same effect was found when we compared TM-ILP patients to patients who underwent amputation: there was no significant difference in global health status/QoL between these two patient groups. Indeed, Zahlten-Hinguranage et al. [23, 42] were also unable to identify differences in QoL between patients with amputation and patients with limb-sparing therapy. Again, the response shift phenomenon is one possible explanation for this effect.

Nonetheless, we identified a difference in QoL specifically between patients with TM-ILP and the EORTC’s all cancer patients group: patients after TM-ILP showed significantly higher scores on the global health status/QoL scale. High-grade soft-tissue sarcomas are highly malignant. The rate of metastasis and, thus, the disease-specific mortality for deep-seated high-grade STS can be 50% or higher [30, 43]. Thus, the patient’s QoL can be reduced significantly, especially in more advanced stages of the disease [13]. To the contrary, our data indicate that the overall QoL of patients after TM-ILP seemed less affected than cancer patients in general.

Regarding the SMFA, which focuses more on the assessment of functional aspects, we found that patients after TM-ILP and tumor resection had higher scores on the dysfunction index, the bother index, and the daily activity and mobility scores. All these results indicate higher degrees of functional problems than in the general population. These findings seem to confirm the results found in earlier studies on the functional scales of the SF-36 [19–21] and indicate that (i) impairment of limb function occurs in sarcoma patients and that (ii) the functional scales of the SMFA seem less prone to the response shift phenomenon than the global health status/QoL score of the QLQ-C30.

Concerning QoL in patients with long-term survival, this study indicated that there was no time dependency of the QoL or function scores, suggesting that patients’ QoL was stable a few months after the end of the local treatment. Even after successful limb salvage, TM-ILP-treated patients with long-term survival after STS do have noticeable problems [21], whereas patients with amputations after STS treatment have more problems and an even lower QoL, as Thijssen et al. [21] demonstrated. This study however, did not reveal any signs of later deterioration of patients’ QoL after TM-ILP.

The strength of this study lies in the use of two validated scores for measuring patients’ QoL after TM-ILP treatment. One of the drawbacks of this study is its selection bias and the rather small number of patients who actually met the inclusion criteria. The use of *t* statistics was necessary in this setting for the comparison to the reference values of the EORTC and amputation groups, but this approach was certainly not ideally suited to this purpose. *T* statistics can be calculated with sole knowledge of the means, variance, and number of cases. The more appropriate non-parametric tests require raw data, which were not available to us from the reference groups. For a further analysis of this matter, we would certainly plan to evaluate a larger number of patients, ideally in a longitudinal study design, combined with a comparison to an existent group of patients who had received different treatment regimens.

In the end, the answer is simple: “Life before limb, but limb is life, too…” [44]. When asked before the procedure, more than 90% of all sarcoma patients would decide against an amputation whenever possible.

## Conclusions

In conclusion, we found that patients with STS after TM-ILP, in combination with complete tumor resection as part of a multidisciplinary limb-sparing approach, showed functional limitations compared to the general population. However, most likely due to the response shift phenomenon, no difference in overall QoL between TM-ILP patients and the general population or patients after amputation was identified in this study setting. TM-ILP patients, however, did exhibit a higher QoL than patients with cancer in general.

Additionally, we found that there was no time dependency of the QoL measures and the end of treatment, suggesting that later deterioration of limb function after TM-ILP and complete tumor resection would not be expected. Further studies in this regard should be pursued, ideally in a longitudinal study design.

## Additional file

Additional file 1: Technical details of TM-ILP. (PDF 307 kb)

## Abbreviations

CI: Confidence interval
ILP: Isolated limb perfusion
MPNST: Malignant peripheral nerve sheath tumor
N: Number of cases
QoL: Quality of life; in this case, health-related quality of life
SD: Standard deviation
SMFA: Short Musculoskeletal Functional Assessment
SMFA-D: German adaption of the SMFA
STS: Soft-tissue sarcoma
TM-ILP: TNF-alpha and melphalan-based isolated limb perfusion (used synonymously with ILP in this text, as all ILPs were TNF- and melphalan-based)
