# Supplementary material for: Lower limb function and quality of life after ILP for soft-tissue sarcoma
Source: World J Surg Oncol. 2017 Apr 13;15:84. doi: 10.1186/s12957-017-1150-3 (PMC5390463; doi:10.1186/s12957-017-1150-3)
Supplement: Additional file 1: — Technical details of TM-ILP. (PDF 307 kb) [file 12957_2017_1150_MOESM1_ESM.pdf]

Technical details of TM-ILP

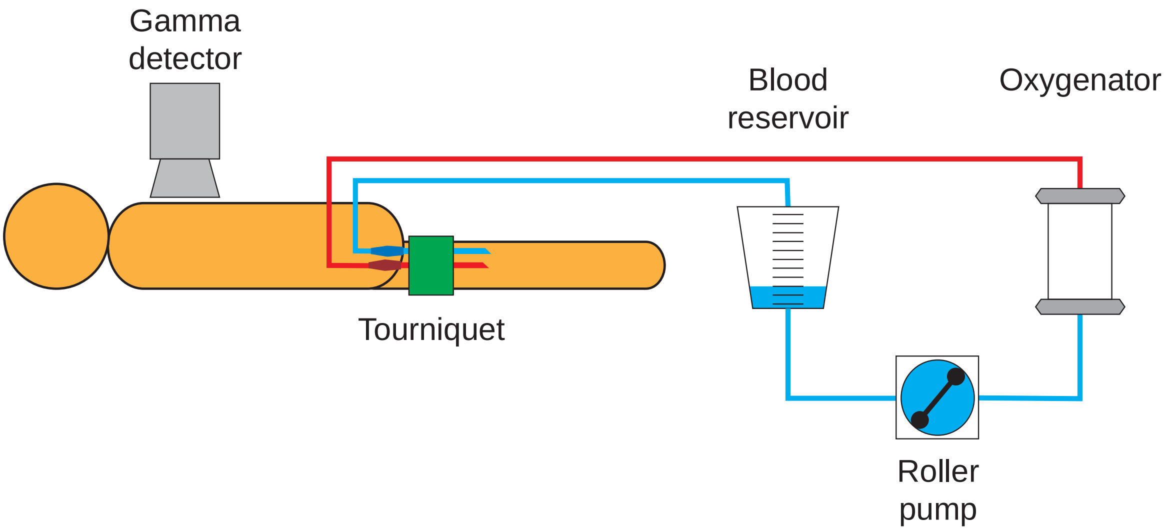

Figure: Schematics of TM-ILP setup. The outline shows the extracorporeal circulation during the procedure in the OR with the arterial and venous cannulae; the heart lung machine with reservoir, pump and oxygenator; and the tourniquet (green) separating the systemic and the extremity circulation. The gamma probe for monitoring leakage was placed above the patient's chest.

Proximal vascular access was established at the iliac level for proximal tumors (located at the thigh) or at the femoral level for tumors located distal to the knee (as displayed above). Perfusion catheters (12 to 16 Fr., Sorin Group, Milan, Italy) were placed into the artery and the vein and connected to a heart lung machine (Stöckert S-III, Sorin Group, Milan, Italy) equipped with a modified, single-circuit perfusion system (Sorin Group, Milan, Italy). For femoral-level treatment, a pneumatic tourniquet was applied proximal to the tips of the catheters; for iliac-level treatment, a doubled 12 cm rubber Esmarch bandage wrapped tightly around the groin was used to temporarily block collateral blood flow from the limb. The extremity was heated to 39°C. After confirmation of successful isolation of the extremity by injection of indium 111-labeled albumin into the extremity circulation and measuring central gamma counts using a gamma probe placed above the heart, 2 mg of TNF-alpha (Beromun, Boehringer-Ingelheim, Ingelheim, Germany) was injected into the isolated extremity. After 15 minutes, melphalan was added at a dose of 11 mg per liter of limb volume (measured according to the protocol described previously) [1]. After another 60 minutes of perfusion, the TNF-melphalan-contaminated blood from the extremity was rinsed with 4-6 liters of colloid solution (Tetraspan 10 %, B. Braun, Melsungen, Germany), the catheters were removed and the vessels were sutured.

1. Podleska LE, Poeppel T, Herbrik M, Dahlkamp L, Grabellus F, Taeger G. Drug dosage in isolated limb perfusion: evaluation of a limb volume model for extremity volume calculation. World J Surg Oncol. 2014; 12: 81.
